# Optimization of biomarkers-based classification scores as progression-free survival predictors: an intuitive graphical representation

**DOI:** 10.4155/fsoa-2018-0020

**Published:** 2018-11-01

**Authors:** Marian Manciu, Sorour Hosseini, Teresa Di Desidero, Giacomo Allegrini, Alfredo Falcone, Guido Bocci, Robert A Kirken, Giulio Francia

**Affiliations:** 1 Department of Physics, University of Texas at El Paso, El Paso, TX, 79968, USA; 2 Division of Pharmacology, Department of Clinical & Experimental Medicine, University of Pisa, Pisa, Italy; 3 Division of Medical Oncology, Pontedera Hospital, Azienda USL of Pisa, Pontedera, Italy; 4 Oncology Unit 2, University Hospital of Pisa, Pisa, Italy; 5 Border Biomedical Research Center, University of Texas at El Paso, El Paso, TX, 79968, USA

**Keywords:** biomarkers, classification scores, prediction of cancer survivability, progression-free survival, survival analysis

## Abstract

**Aim:**

To construct classification scores based on a combination of cancer patient plasma biomarker levels, for predicting progression-free survival.

**Methods:**

The approach is based on the optimization of the biomarker cut-off values, which maximize the statistical differences between the groups with values lower or larger than the cut-offs, respectively. An intuitive visualization of the quality of the classification score is also proposed.

**Results:**

Even if there are only weak correlations between individual biomarker levels and progression-free survival, scores based on suitably chosen combination of three biomarkers have classification power comparable with the Response Evaluation Criteria in Solid Tumors criteria classification of response to treatments in solid tumors.

**Conclusion:**

Our approach has the potential to improve the selection of the patients who will benefit from a given anticancer treatment.

## Lay abstract

A method to construct classification scores based on a combination of cancer patients plasma biomarkers levels, for predicting progression-free survival, is suggested. We show that even if there are only weak correlations between individual biomarker levels and progression-free survival, scores based on a suitably chosen combination of three biomarkers have classification power comparable with the Response Evaluation Criteria in Solid Tumors criteria classification of response to treatments in solid tumors. Since the former classification is derived from data available before the start of treatment, our approach has the potential to improve the selection of patients who will benefit from a given anticancer treatment.

## Graphical abstract

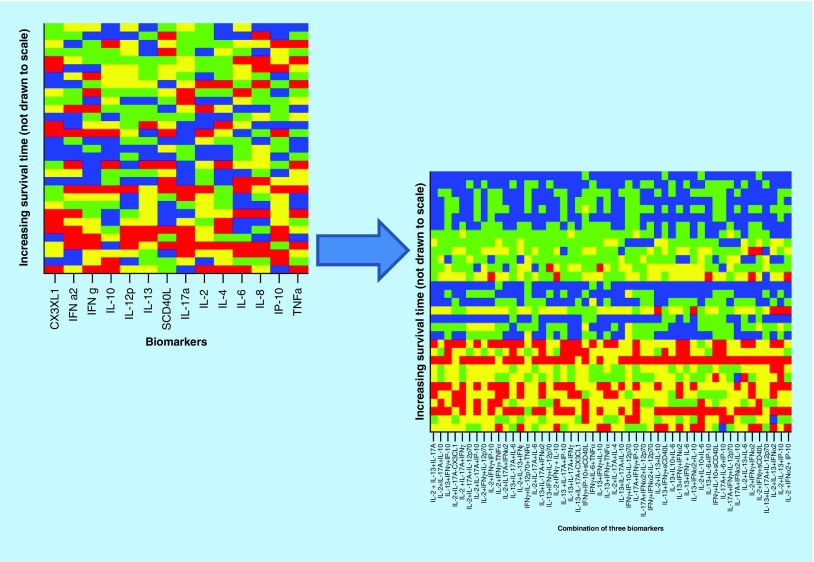

It is well known that some biomarker values strongly correlate with the probability of the onset of diseases or with treatment outcomes; common examples are impaired fasting glycemia, which is correlated with the probability of developing diabetes or high blood pressure values, which are themselves correlated with the probability of developing cardiovascular diseases. Recent advances in high-throughput analysis have tremendously increased the number of potential biomarkers that can be used for the prediction of the onset of diseases or of the treatment outcome. Among the many correlations reported, Van de Vijver *et al*. [1] showed that gene-expression profile is a more powerful predictor of the outcome of disease in young patients with breast cancer than clinical and histological criteria [1]; Llovet *et al*. reported that some plasma biomarkers (Ang2 and VEGF) are independent predictors of survival of patients with advanced hepatocellular carcinoma [2]; Salomaa *et al*. suggested 31 novel biomarkers as predictors for clinically incident diabetes [3]; and Higashimoto *et al*. found out that serum CRP and MMP-9 levels were related to rapid decline in lung function in chronic obstructive pulmonary disease [4].

However, much of the time the correlations between biomarker values and the time-to-event (e.g., onset of the disease, progression-free survival [PFS], tumor relapse, and so forth) are not obvious. Standard statistical correlation methods (Pearson, Spearman) might show very weak (or almost no) correlations between them. The purpose of this article is to suggest a method for optimizing the classification power of a combination of biomarkers, by finding the cut-off values for biomarkers, which maximize the statistical differences between the groups. The main advantages of using a combination of biomarkers (instead of a single one) are twofold: first, the method is more robust, the error in measurement of one biomarker value affecting much less the final classification; second, the method has a much stronger classification power. The method is a straightforward extension to many variables of the optimal cut-off determination (for one variable) based on the most significant split between data, namely the cut-off for which the smallest p-value for the Cox proportional hazard model can be obtained [5].

There are many known statistical learning algorithms to employ multiple variables as predictors, such as Nearest-Neighbors Classifiers, Neural Networks or Support Vector Machines [6]. However, the internal mechanism of selecting the variables and their ranges is somewhat obscure to the biomedical researcher, and therefore many authors prefer to employ the one variable Cox-proportional hazard model as a classifier, which is intuitively more appealing [4]. Furthermore, whereas statistical learning algorithms are extremely efficient for large datasets (for which large training and validation sets can be established), they are less useful for small datasets, such as preliminary data [6]. The method suggested here preserves the intuitive appeal of the one-variable Cox proportional hazard method.

Another issue is that for the optimal selection of the most relevant variables, there are already many performant algorithms (such as Stepwise Optimization, Best Subset, LASSO or Elastic Net [7]). Since our dataset is small (and the general suggestion is that at most about three independent variables should be employed as predictors for its size [7]), the selection of variables in our method is performed *post hoc*. This has the advantage of providing many possible parameter combinations that have a reasonable good classification power. In many realistic situations, the measurement of the ‘optimal’ variables might not possible and viable alternatives have to be sought.

The procedure will be illustrated with existing immune system multiplex data (a 14 cytokine profiling of plasma samples from 31 gastrointestinal cancer patients available at the beginning of a metronomic chemotherapy Phase II clinical trial [8]). Metronomic chemotherapy can be defined as the frequent, regular administration of chemotherapeutic drug doses that maintain a low, prolonged and active range of plasma concentrations of drugs producing a favorable toxicity profile [9]. Thus, a goal of this therapy in the palliative setting is to maintain a SD for a long period of time with a very good tolerability of the given treatments. We show that a score derived from a combination of three biomarkers measured prior to the start of treatment gives approximately the same classification for PFS prediction as the Response Evaluation Criteria in Solid Tumors (RECIST) [10], namely SDs/progressive diseases (PDs), which is normally assessed at a later time point – reducing the time available for further therapeutic intervention.

The suggested approach has the potential to improve the identification of patients who are likely to benefit from the metronomic treatment, and to identify those for whom an alternative therapy might be preferable, therefore reducing both rates of overtreatment or undertreatment.

## Materials & methods

### Clinical data

An immune system multiplex 14-cytokine profiling has been carried out [8] for 31 out of the 38 patients with advanced refractory gastrointestinal tumors of a Phase II clinical trial of metronomic UFT (a 5-fluorouracil prodrug, 100 mg/twice per day per orem [p.o.]) and CTX (cyclophosphamide, 500 mg/mq2 intravenous [iv.] bolus on day 1 and then 50 mg/day p.o.) plus celecoxib [8]. Experimental details as well as a discussion on the implications of cytokines levels for understanding the mechanism of action of metronomic therapy are provided elsewhere [8,11, Valenzuela P et al., Submitted]. In what follows, we employ only the biomarker values measured from the plasma samples taken before the start of treatment (day 0) and we will show that scores constructed on suitably chosen selections of biomarker combinations and optimal cut-off values are excellent predictors for patient survivability. In the study of Allegrini *et al*. [8], PFS, expressed in months, was defined as the time from the first day of treatment until the first documentation of objective disease progression or death due to any cause, whichever occurred first. The assessment of response was done according to RECIST criteria new guidelines to evaluate the response to treatment in solid tumors, with a CT scan of the chest and abdomen, repeated every 8 weeks until PD [9,10]. Response and progression evaluation was based on investigator-reported measurements.

### Statistical method

Correlations between biomarker levels and survival time have been carried out with the standard Pearson and Spearman methods, which for our data shows a very weak correlation between biomarkers values and PFS. For datasets that would show strong correlation, perhaps traditional statistics method (such as generalized regressions methods [6]) would be more appropriate.

The optimal biomarker cut-off values were obtained by minimizing a penalty function consisting of the p-value of the Cox proportional hazard regression between classification groups. Census has been taken into account in the calculations, and the groups have been tested for census imbalance. For individual biomarkers, the minimization was constrained by the condition that at least a third of the patients belong to one of the two possible classification groups, to avoid the excessive influence of potential biomarker value outliers. For constructing scores based on a combination of biomarkers, the minimum was sought only in the vicinity of individual biomarker cut-offs, to avoid the clustering of too many (or too few) patients in one classification group. Regarding the later scenario, it is fairly easy to identify a few patients, who have the longer PFS, but the classification method is strongly affected by the potential measurement errors or individual variability in the biomarkers values. This is a common problem in statistical learning, related to the ‘overfitting’ of the data.

The scores (classifications) derived from individual biomarkers are 0 or 1 if the biomarker value is less or equal or larger than the cut-off value, respectively, (it is the opposite for the biomarkers that are negatively correlated with the survivability, namely IL-6, IL-12, IP-10 and IL-8) The scores derived from combinations of biomarkers are calculated as the sum of the individual biomarkers scores.

From an intuitive point of view, the classification score can be associated with a color level: if the color levels are properly chosen, one would expect some color (e.g., blue) to be well correlated with long survival times and other color (e.g., red) to be well correlated with short survival times. As it will be shown in what follows, an unoptimized guess for the ‘color levels’ results in a picture with no apparent correlation between ‘color’ and PFS. Optimal cut-off values and the sorting of the individual biomarkers based on the lowest p-values resulted in much better correlations. For scores based on biomarker combinations, among the 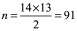 possible combinations of two biomarkers, only the best 27 (with p < 0.01) are represented in the picture; among the 
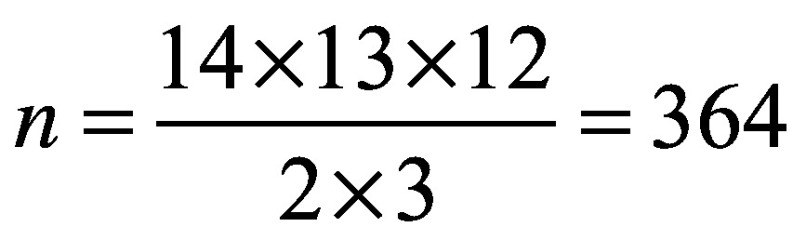
 possible combinations of two biomarkers, only the best 50 are represented (with p < 0.0023).

It should be emphasized that the resulting value for p is an indication of the quality of the biomarkers and cut-off selections, but does not directly represent the probability that the result is due to chance (which is affected by multiple-possibility of cut-offs and biomarkers selections).

All the calculations are performed with our proprietary MATLAB codes, which are available upon request from the corresponding author.

## Results & discussion

**Figure 1.**
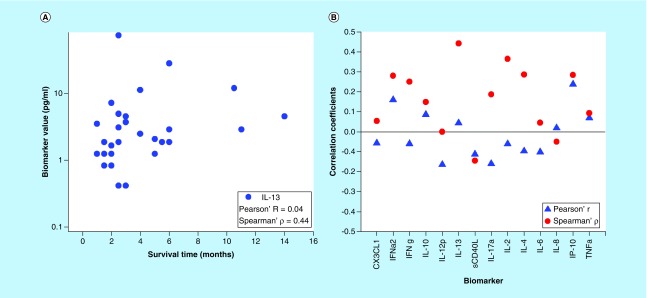
**Correlations between biomarker values and survival.** **(A)** Values of IL-13 as a function of the survival time. **(B)** Pearson and Spearman correlation coefficients between biomarker values and survival time for individual biomarkers.

In general, in our multiplex experiment, plots of biomarker values as functions of the PFS time do not suggest a good correlation between them (Figure 1A shows the values of IL-13 vs survival time, the biomarker that exhibit the largest Spearman's correlation coefficient from the set).

The values of the standard correlation coefficients, Pearson's r and Spearman's ρ, are plotted in Figure 1B for each of the 14 biomarkers investigated. Small values of the Pearson's r coefficient 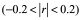 are in general associated with no (or negligible) correlations. When a linear relationship between biomarker values and survival time is not expected, Spearman coefficient (which addresses only the existence of a monotonic relationship) is perhaps more appropriate. Indeed, for our dataset, it will be shown in what follows that the proposed method indicates that the best single biomarkers predictors are IL-2, IL-13, IL-17α, IFN-γ and IFN-α2, which are consistent with the large values of the Spearman's ρ coefficient from Figure 1B. Also, it will turn out that IL-6, IL-12p and IL-8 (with some of the lowest ρ coefficients) are inversely correlated with survival.

**Figure 2.**
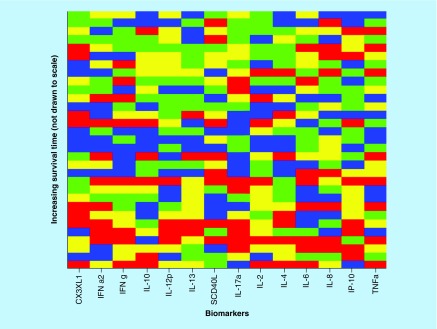
**Quartiles cut-offs for individual biomarker values show no apparent association with the survival time.** For strong correlations, the top of the picture would be mostly blue and the bottom mostly red (first quartile = red; second quartile  = yellow; third quartile = green; forth quartile = blue).

One intuitive way to visualize the correlation between biomarkers and survivability is to associate a score (color level) with their values. When the cut-offs for the scores are based on the quantile values for the biomarkers, there is no obvious association, as shown in Figure 2. The patients are sorted in the order of increasing survival time along the vertical axis, and for strong correlations, the top of the picture would be mostly blue and the bottom mostly red.

**Figure 3.**
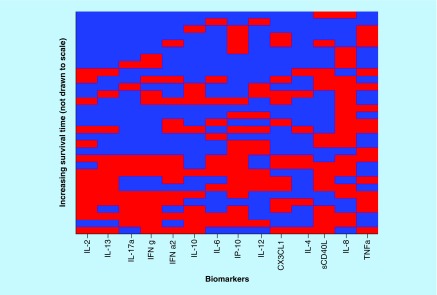
**Optimized cut-offs for individual biomarker values.** For strong correlations, the top of the picture would be mostly blue and the bottom mostly red; along the horizontal direction, the biomarkers are sorted in the order of decreasing predictive power (score = 0, red; score = 1, blue).

After optimization of the biomarker cut-offs and sorting the biomarkers in the order of decreasing statistical significance along the horizontal axis, Figure 3 was obtained.

The biomarkers in Figure 3 are ordered in decreasing predictive power, which is recorded in Table 1, as well as the p-values between the Kaplan–Meier estimators of the two groups selected based on the optimal cut-offs and their corresponding hazard ratios. Only for the first five biomarkers are there statistically significant differences between the groups with individual biomarker values lower or larger than the optimal cut-offs, respectively.

**Table 1.** **Biomarkers ordered in decreasing predictive power.**

**X coordinate**	**Biomarker**	**p-value**	**λ (hazard ratio)**
1	IL-2	0.0080	3.183

2	IL-13	0.0087	3.177

3	IL-17A	0.018	2.837

4	IFN-γ	0.023	2.584

5	IFN-α2	0.044	2.279

6	IL-10	0.064	2.091

7	IL-6	0.13	1.831

8	IP-10	0.16	1.767

9	IL-12p70	0.19	1.690

10	CX3CL1	0.21	1.644

11	IL-4	0.23	1.778

12	sCD40L	0.25	1.553

13	IL-8	0.30	1.573

14	TNFα	0.47	1.325

X coordinate is from Figure 3.

**Figure 4.**
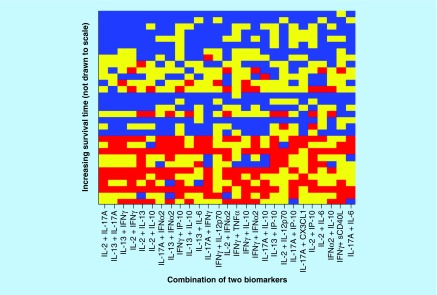
**Optimized cut-offs for combinations of two biomarker values.** For strong correlations, the top of the picture would be mostly blue and the bottom mostly red; along the horizontal direction, the biomarkers are sorted in the order of decreasing predictive power (score = 0, red; score = 1, yellow; score = 2, blue).

For a combination of two biomarkers (see Figure 4 & Table 2), there are 27 combinations with p < 0.

**Table 2.** **Combinations of two biomarkers ordered in decreasing predictive power.**

**X coordinate**	**Biomarker**	**Biomarker**	**p-value**
1	IL-2	IL-17A	0.00064

2	IL-13	IL-17A	0.0015

3	IL-13	IFN-γ	0.0018

4	IL-2	IFN-γ	0.0020

5	IL-2	IL-13	0.0027

6	IL-2	IL-10	0.0033

7	IL-17A	IFN-α2	0.0040

8	IL-13	IFN-α2	0.0041

9	IFN-γ	IP-10	0.0047

10	IL-13	IL-10	0.0049

11	IL-13	IL-6	0.0057

12	IL-17A	IFN-γ	0.0064

13	IFN-γ	IL-12p70	0.0064

14	IL-2	IFN-α2	0.0066

15	IFN-γ	TNFα	0.0074

16	IFN-γ	IL-10	0.0075

17	IFN-γ	IFN-α2	0.0079

18	IL-17A	IL-10	0.0083

19	IL-13	IP-10	0.0084

20	IL-2	IL-12p70	0.0090

21	IL-17A	IP-10	0.0092

22	IL-17A	CX3CL1	0.0094

23	IL-2	IP-10	0.0098

24	IL-2	IL-6	0.0100

25	IFN-α2	IL-10	0.0101

26	IFN-γ	sCD40L	0.0103

27	IL-17A	IL-6	0.0105

X coordinate is from Figure 4.

**Figure 5.**
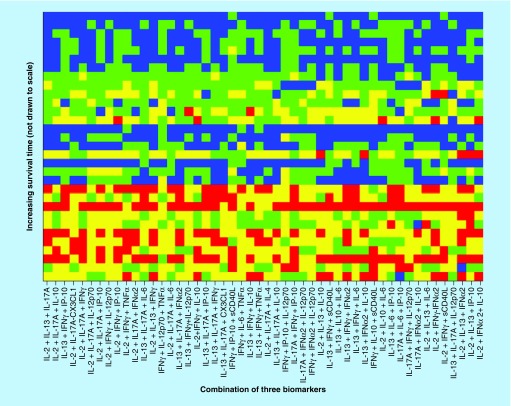
**Optimized cut-offs for combinations of two biomarker values.** For strong correlations, the top of the picture would be mostly blue and the bottom mostly red; along the horizontal direction, the biomarkers are sorted in the order of decreasing predictive power (score  = 0, red; score = 1, yellow; score = 2, green; score = 3, blue).

Finally, the scores obtained for the best 50 combinations of three biomarkers are illustrated in Figure 5, and the best biomarkers combinations are provided in Table 3.

**Table 3.** **Combinations of three biomarkers ordered in decreasing predictive power.**

**X coordinate**	**Biomarker**	**Biomarker**	**Biomarker**	**p-value**
1	IL-2	IL-13	IL-17A	0.00047

2	IL-2	IL-17A	IL-10	0.00063

3	IL-13	IFN-γ	IP-10	0.00069

4	IL-2	IL-17A	CX3CL1	0.00071

5	IL-2	IL-17A	IFN-γ	0.00072

6	IL-2	IL-17A	IL-12p70	0.00075

7	IL-2	IL-17A	IP-10	0.00077

8	IL-2	IFN-γ	IL-12p70	0.00078

9	IL-2	IFN-γ	IP-10	0.00080

10	IL-2	IFN-γ	TNFα	0.00089

11	IL-2	IL-17A	IFN-α2	0.00091

12	IL-13	IL-17A	IL-6	0.00094

13	IL-2	IL-13	IFN-γ	0.00095

14	IFN-γ	IL-12p70	TNFα	0.00095

15	IL-2	IL-17A	IL-6	0.00095

16	IL-13	IL-17A	IFN-α2	0.00097

17	IL-13	IFN-γ	IL-12p70	0.000100

18	IL-2	IFN-γ	IL-10	0.00105

19	IL-13	IL-17A	IP-10	0.00105

20	IL-13	IL-17A	IFN-γ	0.00105

21	IL-13	IL-17A	CX3CL1	0.00114

22	IFN-γ	IP-10	sCD40L	0.00115

23	IFN-γ	IL-6	TNFα	0.00121

24	IL-13	IFN-γ	IL-10	0.00122

25	IL-13	IFN-γ	TNFα	0.00122

26	IL-2	IL-17A	IL-4	0.00130

27	IL-13	IL-17A	IL-10	0.00130

28	IFN-γ	IP-10	IL-12p70	0.00130

29	IL-17A	IFN-γ	IP-10	0.00135

30	IL-17A	IFN-α2	IL-12p70	0.00138

31	IFN-γ	IFN-α2	IL-12p70	0.00139

32	IL-2	IL-13	IL-10	0.00140

33	IL-13	IFN-γ	sCD40L	0.00148

34	IL-13	IL-10	IL-6	0.00150

35	IL-13	IFN-γ	IFN-α2	0.00160

36	IL-13	IFN-γ	IL-6	0.00179

37	IL-13	IFN-α2	IL-10	0.00189

38	IFN-γ	IL-10	sCD40L	0.00191

39	IL-2	IL-10	IL-6	0.00194

40	IL-13	IL-6	IP-10	0.00195

41	IL-17A	IL-6	IP-10	0.00207

42	IL-17A	IFN-γ	IL-12p70	0.00209

43	IL-17A	IFN-α2	IL-10	0.00210

44	IL-2	IL-13	IL-6	0.00210

45	IL-2	IFN-γ	IFN-α2	0.00210

46	IL-2	IFN-γ	sCD40L	0.00214

47	IL-13	IL-17A	IL-12p70	0.00224

48	IL-2	IL-13	IFN-α2	0.00225

49	IL-2	IL-13	IP-10	0.00227

50	IL-2	IFN-α2	IL-10	0.00229

X coordinate is from Figure 5.

As perhaps expected, the best three individual biomarkers predictors for survivability, namely IL-2, IL-13 and IL-17α, contribute also to the best predictor score based on the combination of three biomarkers. However, there are many other biomarkers combinations, which are almost as good as survivability predictors.

**Figure 6.**
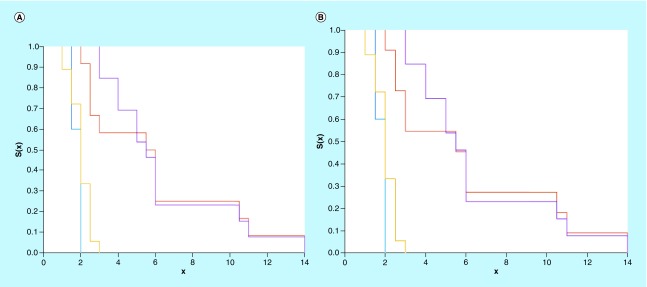
**Kaplan–Meier survival estimators.** **(A)** progressive disease group (yellow); stationary disease group (purple); lowest score based on IL-2, IL-13 and IL-17α values (blue); highest score based on IL-2, IL-13 and IL-17α values (red). **(B)** progressive disease group (yellow); stationary disease group (purple); lowest score based on IFN-γ, IL-12p and TNFα values (blue); highest score based on IFN-γ, IL-12p and TNFα values (red).

The evaluation of the predictive power of the scores based on three-biomarker combinations is performed by comparing the groups with best and worst scores to the RECIST criteria classification ‘SD/PD’ [10,12], as shown in Figure 6A. There are no statistically significant differences between the SD classification (purple) and higher score group (red), or between the PD classification (yellow) and the lowest score group (blue).

Interestingly, the score classification based on a combination of biomarkers has a strong predictive power even when the individual biomarkers are not well correlated with PFS; for example, in Figure 6B the ‘PD/SD’ classification is compared to the groups of highest and lowest scores, derived from the values of IFN-γ, IL-12p and TNFα, which are the forth, the ninth and the worst individual predictors for survivability, respectively (out of the 14 biomarkers investigated; see Table 1).

## Conclusion

The ability to predict the likelihood of the onset of the disease, or the response a treatment, is important in oncology. Recent advances in technology have tremendously increased the number of available biomarkers that can be used as potential predictors. However, many of the biomarkers do not (at least apparently) correlate well with the event that has to be predicted (in this case, PFS).

A method to derive classification scores, based on a suitably chosen combination of biomarkers, was suggested. It was shown that some of the classification scores might be very good predictors of the PFS, even when the individual biomarkers do not exhibit strong correlations with it. Our method is based on finding the optimal cut-offs in biomarkers values, which provide the smallest p-values between the classification groups. This is a straightforward extension to many dimensions of the optimal cut-off determination for one variable, via the minimization of the p-score of the Cox proportional hazard. An intuitive graphical visualization of the quality of the scores was also proposed.

For the data of an immune system multiplex 14-cytokine profiling of plasma samples available from 31 patients with advanced refractory gastrointestinal tumors [8,11] ([Valenzuela P et al., Submitted), we showed that the scores derived from the suitably chosen combinations of three biomarkers has a classification power comparable to the RECIST criteria clinical classification. Since (unlike the SD/PD evaluation that was performed every 8 weeks of treatment), the samples were collected before the beginning of the treatment, the classification power of the suggested method has the potential to lead to a better selection of the patients that will mostly (or least) benefit from the treatment. An additional advantage of the method is that it provides many possible combinations of the parameters with good classification power, therefore, future experiments can select the combination of biomarkers that are available.

## Future perspective

The probability of the onset of diseases or the treatment outcome has been correlated in traditional medicine with the values of a few biomarkers, which have been easily accessible (such as the body temperature). The recent tremendous increase in the number of available biomarkers as well as development in artificial intelligence will lead in the near future to the development of personalized medicine, in which particularities of the patients will dictate the optimal treatment. Our method to select the most reliable parameters (among the available set) and consequently to classify the patient into a group that benefits the most from the given treatment is not restricted to cancer, but has broader applications in medical treatment.

**Summary points**

- It is well known in traditional medicine that a few biomarkers are typically well correlated with the probabilities of developing a disease or with the outcome of the treatment.
- Recently, the number of available biomarkers has been increasing (e.g., differential gene expressions), although it might not be clear which biomarkers are correlated with the desired outcome.
- We showed that even if individual biomarkers are not well correlated with the outcome (in the traditional statistical sense, namely Pearson or Spearman correlations), a suitable selected combination of parameters has a strong classification power.
- A method of constructing combinations of biomarkers is presented, based on the optimization of the biomarker cut-off values, which maximize the statistical differences between the groups with values lower or larger than the cut-offs, respectively.
- An intuitive visualization of the quality of the classification score is also proposed.
- We used available data for 14 cytokines values and demonstrated that suitably chosen combination of three biomarkers have classification power comparable with the Response Evaluation Criteria in Solid Tumors criteria classification of response to treatments in solid tumors.
- Since the former classification is derived from data available before the start of treatment, our approach has the potential to improve the selection of the patients who will benefit from a given anticancer treatment.
- Our method has general applicability in predicting outcomes based on initial value of the parameters, is scalable to a large number of parameters, and therefore might have broader applications.
